# Antiviral and Virucidal Activities of N**α**-Cocoyl-L-Arginine Ethyl Ester

**DOI:** 10.1155/2011/572868

**Published:** 2011-11-28

**Authors:** Hisashi Yamasaki, Kazuko Tsujimoto, Keiko Ikeda, Yukiko Suzuki, Tsutomu Arakawa, A. Hajime Koyama

**Affiliations:** ^1^Division of Virology, Department of Cellular and Molecular Medicine, Graduate School of Medicine, Wakayama Medical University, 580 Mikazura, Wakayama 641-0011, Japan; ^2^School of Health and Nursing Science, Wakayama Medical University, 580 Mikazura, Wakayama 641-0011, Japan; ^3^Alliance Protein Laboratories, Thousand Oaks, CA 91360, USA

## Abstract

Various amino acid-derived compounds, for example, N**α**-Cocoyl-L-arginine ethyl ester (CAE), alkyloxyhydroxylpropylarginine, arginine cocoate, and cocoyl glycine potassium salt (Amilite), were examined for their virucidal activities against herpes simplex virus type 1 and 2 (HSV-1 and HSV-2), influenza A virus (IAV), and poliovirus type 1 (PV-1) in comparison to benzalkonium chloride (BKC) and sodium dodecylsulfate (SDS) as a cationic and anionic control detergent and also to other commercially available disinfectants. While these amino acid-derived compounds were all effective against HSV-1 and HSV-2, CAE and Amilite were the most effective. These two compounds were, however, not as effective against IAV, another enveloped virus, as against HSV. Cytotoxicity of CAE was weak; at 0.012%, only 5% of the cells were killed under the conditions, in which 100% cells were killed by either SDS or BKC. In addition to these direct virucidal effects, CAE inhibited the virus growth in the HSV-1- or PV-1-infected cells even at 0.01%. These results suggest a potential application of CAE as a therapeutic or preventive medicine against HSV superficial infection at body surface.

## 1. Introduction

Recently there has been an increasing necessity for a multitude of preparedness against infectious diseases. Every year we encounter emerging and reemerging infectious diseases. While vaccination is a primary strategy to overcome viral diseases [1], vaccines are not readily available for many viruses and, even if available, not accessible for every individual due to various reasons (e.g., cost, distribution, and politics). Another compounding problem with virus infection is that antiviral drugs are not available for many viral diseases [2]. Disinfection at body surface, such as hand sanitization, has been an effective measure for the prevention against viral diseases and even applicable for the therapy of certain viral diseases, provided that its tissue toxicity can be tolerated [3].

A number of compounds, including organic solvents and detergents, are known to inactivate viruses [4]. However, most of these disinfectants are generally toxic to cells and tissues, when come in contact with skins and in particular mucosal surfaces, limiting their* in vivo* applications. We have observed that aqueous arginine solution inactivates various enveloped viruses, although relatively high arginine concentration was required [5–10]. Such enhanced virus inactivation by arginine was considered applicable for clearance of contaminated viruses in biopharmaceutical products. Antiviral activity of arginine opened a window for topical applications against superficial infectious diseases where both virucidal and antiviral activities might reduce the viral load in the infected mucosal cells. Here we have undertaken a systematic screening of amino acid derived compounds in search for effective, nontoxic antiviral and virucidal agents, which can be used for both hand sanitization and superficial infections on body surface. Several available compounds were tested for their effects on inactivation of herpes simplex viruses, influenza virus, and poliovirus and their toxic effects on cultured cells and compared with the commercially available known disinfectants.

## 2. Materials and Methods

### 2.1. Cells and Viruses

MDCK, HEp-2, and Vero cells were grown in Eagle's minimum essential medium (MEM) containing 5% fetal bovine serum. Herpes simplex virus type 1, strain F, (HSV-1), herpes simplex virus type 2, strain 186, (HSV-2), influenza virus A/Aichi/68 (IAV) and poliovirus type 1 (PV-1), Sabin vaccine strain, were used throughout the experiments [11–13]. The viruses were propagated in Vero cells (for HSV-1, HSV-2, and PV-1) in MEM supplemented with 0.5% fetal bovine serum (FBS) or in MDCK cells (for IAV) in MEM supplemented with 0.1% bovine serum albumin (BSA) and acetylated trypsin (4 *μ*g/mL). The viruses were stored at −80°C until use. The amount of each virus was measured by a plaque assay as described previously [11–13].

### 2.2. Reagents

L-arginine hydrochloride (referred as arginine), butyroyl-L-arginine (ButArg), N*α*-cocoyl-L-arginine ethyl ester (CAE), N-[3-alkyl(12,14)oxy-2-hydroxypropyl]-L-arginine hydrochloride (Amisafe LMA-60), L-arginine cocoate (Aminosoap AR-12), potassium N*α*-Cocoyl-L-glycinate (Amilite GCK-12K), benzalkonium chloride (BKC), sodium dodecylsulfate (SDS), sodium N*α*-dodecanoylsarcosinate (LS), Chlorhexidine gluconate (CHX), ethylalchol (EtOH), povidone iodine (PI), and sodium hypochlorite (NaOCl) were of biochemical research grade and kindly provided by Ajinomoto Co., Inc (Tokyo, Japan). The compounds with trademark are used as ingredients for cosmetic products. The pH of each solvent was adjusted to the desired values using pH electrode type 6378 and pH meter type F-54 (Horiba; Kyoto, Japan). A pH meter was manually calibrated every 2 h during the preparation of test solutions.

### 2.3. Assay for Virucidal Activity

All the starting materials were stored on ice prior to the virus inactivation experiments. An excess volume of solutions was mixed with the virus stock so that both the pH and concentration of the test solutions would not be affected: that is, a 190 *μ*L aliquot of the solutions received a 10 *μ*L aliquot of virus preparations (approximately 10^7^ ~ 10^8^ plaque-forming units [PFU]/mL). This virus preparation was incubated at the indicated temperature for 5 min. After incubation, the test mixtures were chilled in ice-water bath, and, immediately, aliquots of these virus samples were 100-fold diluted with ice-cold Dulbecco's phosphate-buffered saline (PBS) without Ca^2+^ and Mg^2+^ containing 0.1% BSA for IAV or 0.5% FBS for other viruses to stop the virus inactivation. The viruses were further diluted to yield virus counts suitable for measurements, and the number of infectious virus in the treated preparation was measured by a plaque assay on Vero (for HSV-1, HSV-2, and PV-1) or MDCK (for IAV) cells. There was little virus inactivation in PBS, and hence the amount of infectious virus in PBS was used as a control count [14].

### 2.4. Effect of the Reagent on the Multiplication of the Virus

Monolayered cells in 35 mm dishes were infected with the virus at an indicated multiplicity of infection (MOI). The infected cells were further incubated at 37°C (for HSV-1, HSV-2, and IAV) or 35.5°C (for PV-1) for the indicated period in the serum-free MEM (pH 7.4) containing 0.1% BSA and the indicated concentrations of the test compounds. The compounds were added at the onset of the incubation. For the experiments with influenza virus, acetylated trypsin (4 *μ*g/mL) was also added to the medium for the proteolytic activation of virus infectivity. At the indicated time, the culture medium was harvested and the amount of total progeny virus in the culture was determined as described previously [11–13].

### 2.5. Assay for the Cytotoxicity

Confluent monolayers of Vero cells were incubated in 0.85% sodium chloride solution (pH 5.5) containing 20 mM MES (2-Morpholinoethanesulfonic acid) and varying concentrations of each test compound at room temperature for 15 min. To determine the extents of cell death, monolayered cells were trypsinized to obtain single cell suspension. After the addition of MEM containing 10% calf serum to the suspension to neutralize the trypsin and to stabilize the cells, the number of the living or dead cells was determined by a dye exclusion method with trypan blue as described previously [15].

## 3. Results and Discussion

### 3.1. Effect of Amino Acid Derived Compounds on the Infectivity of DNA and RNA Viruses

We examined the virucidal activity of arginine and amino acid derived compounds (i.e., 0.5 M arginine, 0.5 M ButArg, 0.2% CAE, 0.2% Amisafe, 0.2% Aminosoap, and 0.2% Amilite) on viruses of three completely different kinds; that is, HSV-1 and HSV-2 (Family of Herpesviridae), IAV (Family of Orthomyxoviridae), and PV-1 (Family of Picornaviridae). Both HSVs and IAV are large enveloped viruses and need the cell nucleus for the virus multiplication: the former has a double-stranded DNA genome, and the latter has a segmented negative-stranded RNA genome [16, 17]. In contrast to the viruses of these two virus families, PV-1 is a small nonenveloped virus carrying a positive-stranded RNA as a genome and replicates in the cytoplasm of the infected cells [18].

**Figure 1:**
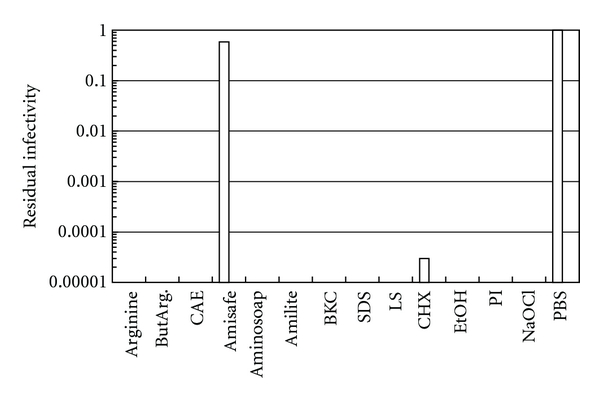
Inactivation of HSV-1. The virus was incubated in the solution indicated below for 5 min at 30°C. Number of infectious virus was determined by a plaque assay on Vero cells after the incubation and was normalized to that incubated in PBS. Arginine (0.5 M at pH 4.0 in 10 mM citrate), ButArg (0.5 M in pH 4.0 in 10 mM citrate), CAE (0.2% in 5 mM citrate and 0.15 M NaCl at pH 4.8), Amisafe (0.2% in 5 mM citrate and 0.15 M NaCl at pH 4.8), Aminosoap (0.2% in 5 mM citrate at pH 7.0), Amilite (0.2% in 5 mM citrate at pH 8.0), BKC (0.1% in 5 mM citrate at pH 7.0), SDS (0.2% in 5 mM citrate at pH 7.0), LS (0.2% in 5 mM citrate at pH 7.0), CHX (0.5% at pH 6.0), EtOH (70% in 5 mM citrate at pH 7.0), PI (0.23% in 5 mM citrate at pH 3.5), NaOCl (100 ppm in 5 mM phosphate at pH 7.0), and PBS (pH 7.4).

Figure 1 shows the effects of these compounds on the infectivity of HSV-1, when the virus was incubated at 30°C for 5 min. As a control, the virus was incubated in PBS and the relative residual infectivity for each compound was calculated by dividing the number of the infectious virus in the test solutions by that of the PBS control. As shown in Figure 1, HSV-1 was sensitive to these compounds except for Amisafe; the virus infectivity decreased below or near the detection level (10^−5^ of the control). In the case of Amisafe, the decrease in the virus infectivity was marginally less than that incubated in the PBS. These results clearly show that arginine and amino acid derived compounds (except Amisafe) can efficiently inactivate HSV-1, an enveloped DNA virus, under the experimental conditions. Although pH, ion species, or ionic strength of the solution can affect the degree of virus inactivation [14], we have shown before that HSV-1 is relatively stable above pH 4.8 and the effects of pH 4.0 alone are much weaker than the combined effects of the pH and arginine or ButArg [5, 6]. The results were compared with commercial disinfectants (i.e., 0.1% BKC, 0.2% SDS, 0.2% LS, 0.5% CHX, 70% EtOH, 0.23% PI, and 100 ppm NaOCl). These commercial disinfectants also showed strong virus inactivation on HSV-1 (Figure 1), although the effect of PI may be entirely due to the low pH (i.e., pH 3.5). Enveloped viruses are known to be sensitive to detergents, which can perturb and solubilize lipid bilayers in the viral envelope. In agreement with this, HSV-2, another human herpesvirus of different serotype and pathogenicity, also showed a similar sensitivity profile to these amino acid derivatives and disinfectants (data not shown). 

**Figure 2:**
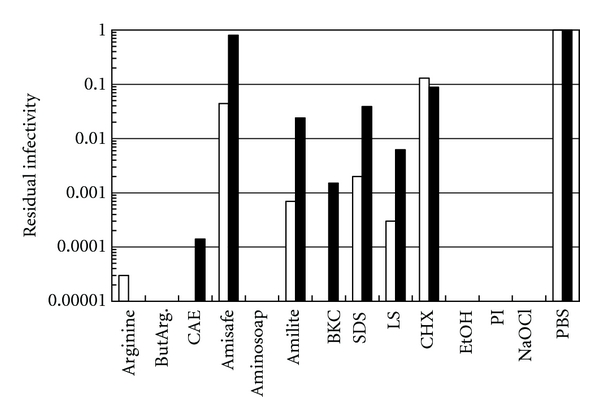
Inactivation of IAV. See the legend to Figure 1. Black and white bars represent the results in the presence or absence of 0.1% BSA.

Next, we tested these compounds in Figure 1 against IAV, an enveloped virus of another virus family. As shown in Figure 2 (white columns), the profile was quite different from that observed for HSV-1 (and HSV-2). ButArg, CAE, Aminosoap, BKC, EtOH, PI, and NaOCl reduced the IAV infectivity below the detection level (10^−5^ of the control). Arginine, Amisafe, Amilite, SDS, LS, and CHX showed a varying degree of virucidal activities, with Amisafe being significantly less effective, similar to its effect on HSV-1, and with CHX being least effective (white columns in Figure 2). We have shown before that the pH profile of IAV inactivation is complex [14] and, at least, a part of inactivation by low pH samples (arginine, ButArg, and PI) is due to the pH, but that the pH alone cannot explain the strong inactivation by arginine and ButArg [5, 6]. Interestingly, the addition of 0.1% BSA resulted in suppression, at least partially, of IAV inactivation by CAE, Amisafe, Amilite, BKC, SDS, and LS (black columns in Figure 2). This is unlikely due to the interaction of BSA with the virus, as it did not affect the virus infectivity in PBS. It is most likely due to the ability of BSA to bind fatty acids [19] and hence these compounds that have nonpolar moieties. 

**Figure 3:**
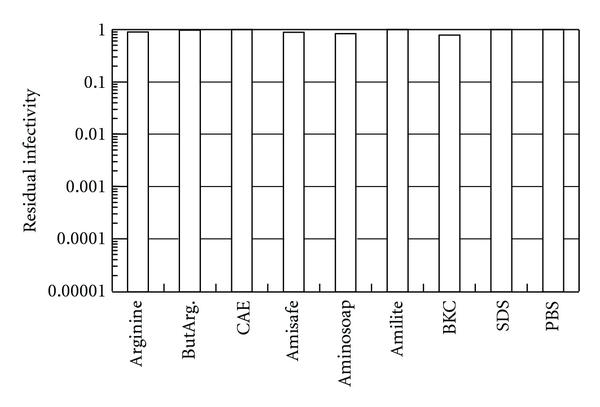
Inactivation of poliovirus. See the legend to Figure 1.

The observed effects of these compounds on IAV suggest the potential mechanism of inactivation. Comparison of arginine, ButArg, CAE, Aminosoap, BKC, EtOH, PI, and NaOCl, which showed strong virucidal effects on this virus, demonstrates no distinct pattern with compound structure. Arginine and ButArg are known to suppress protein-protein interactions, probably through their ability to interact with proteins [20–22]. CAE, Aminosoap, and BKC have surfactant properties. EtOH is an organic solvent. These compounds can interact with not only proteins, but also lipid membranes. PI and NaOCl are strong oxidants and can interact with most components of virus particles. On the contrary, there appears to be a unique structure pattern for less active compounds, that is, Amisafe, Amilite, SDS, LS, and CHX. These are all detergents, meaning that detergent-like properties are insufficient for the effective virus inactivation. However, these detergents are effective against HSV-1, demonstrating that the virucidal effectiveness is virus dependent. In contrast to these enveloped viruses, poliovirus was resistant to inactivation by these amino acid derived compounds, as only negligible degree of the inactivation was observed (Figure 3). Even combination of pH 4.0 and arginine or ButArg was insufficient. The lack of sensitivity of PV-1 may suggest that inactivation of HSV-1 (Figure 1) and IAV (Figure 2) by these compounds requires the presence of lipid envelope on the structure of target viruses. It should be noteworthy that although these compounds appear to act on lipids, it does not necessarily mean solubilization of envelope lipid bilayer. The concentrations of the detergents used in these experiments (0.2%) are below critical micelle concentrations (CMCs). Although the CMCs of ionic detergents are generally affected by the pH of detergent solutions, they are considered to be in the range of ~10 mM (approximately 0.4%) under the experimental conditions. Below CMC, detergents are unlikely capable of destroying membrane structure.

Among these compounds, CAE and Aminosoap showed a marked virucidal activity. Considering that CAE has been reported to have bacteriocidal activity [23], we further characterized the virucidal activity of CAE.

### 3.2. Characterization of Virucidal Activity of CAE

**Figure 4:**
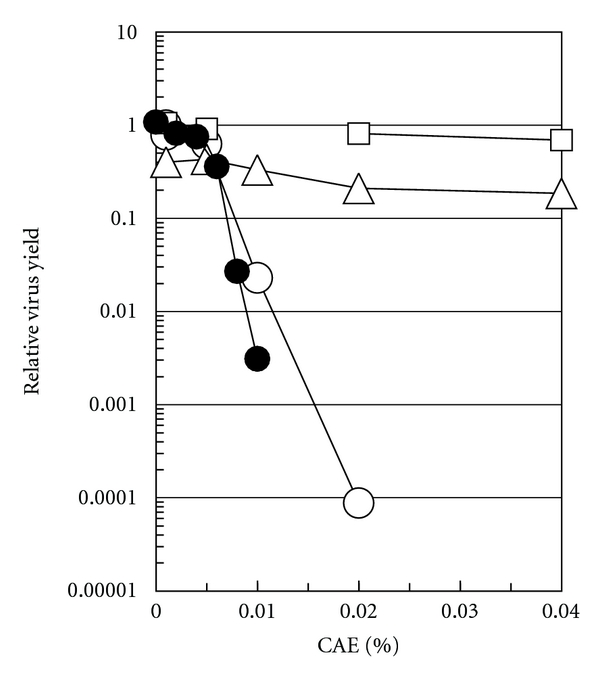
Inactivation of viruses by CAE. The viruses were incubated at 20°C for 5 min in 10 mM citrate buffer (pH 5.0) containing 0.15 M sodium chloride and varying concentrations of CAE. Number of infectious virus was determined by a plaque assay after the incubation and was normalized to that incubated in PBS (pH 7.4). Circles (○) for HSV-1; black circle (●) for HSV-2; triangles (△) for IAV; squares (□) for PV-1.

Figure 4 shows the effects of CAE concentration on the inactivation of HSV-1, HSV-2, IAV, and PV-1. As CAE was more soluble at acidic pH, we used pH 5.0 in these experiments. This pH alone caused little inactivation for HSVs (less than 20% inactivation, if any) and PV-1 (almost no inactivation), but a significant inactivation for IAV (50 ~ 70% inactivation) [14]. This is probably due to acid-induced conformational change of HA spike proteins present in IAV envelope [17]. Under the concentration range (0.001 to 0.04% CAE), PV-1 showed only a marginal loss of the infectivity, while IAV showed a small, but significant (60% decrease at 0.04% CAE), decrease in infectivity with increasing CAE concentrations. In contrast to these two viruses, both HSV-1 and HSV-2 showed a sharp decrease in infectivity, leading to 10^4^-fold reduction at 0.02% CAE for HSV-1 and ~10^3^-fold reduction at 0.01% CAE for HSV-2. Although both HSV-1 and HSV-2 belong to the same virus family, HSV-2 showed a significantly stronger inactivation than HSV-1 at low CAE concentrations. This virucidal activity of CAE was slightly, but significantly, enhanced by lowering the pH of the test solutions to 4.0 or 4.5; for example, the inactivation of HSV-1 or HSV-2 at 0.005% CAE was only marginal at pH 5.0, but was enhanced 10-fold (for HSV-1) or 100-fold (for HSV-2) at pH 4.0 at the same CAE concentration.

**Figure 5:**
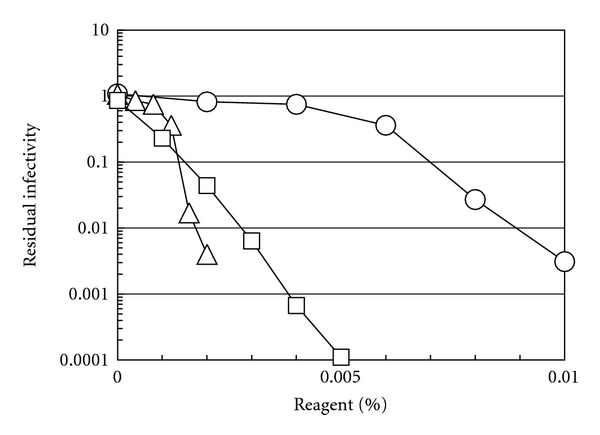
Effects of CAE, BKC, and SDS on the infectivity of HSV-2. HSV-2 was incubated in PBS (pH 7.4) containing varying concentrations of each compound at 30°C for 5 min. The amounts of infectious progeny viruses were determined by a plaque assay after the incubation and were normalized to that incubated in PBS (pH 7.4). Circles (○) for CAE; triangles (△) for BKC; squares (□) for SDS.

To further characterize the virucidal effects of CAE, it was compared with SDS, an anionic detergent, and BKC, a cationic detergent, at neutral pH. Figure 5 shows such a comparison of virucidal activities against HSV-2. Both BKC and SDS showed marked effects (more than two-log reduction) on HSV-2 infectivity even at low concentrations, for example, at 0.002% and 0.003%, respectively, while CAE required higher concentrations to achieve a similar level of inactivation under these conditions.

### 3.3. Cytotoxic Effects of the Reagents

**Figure 6:**
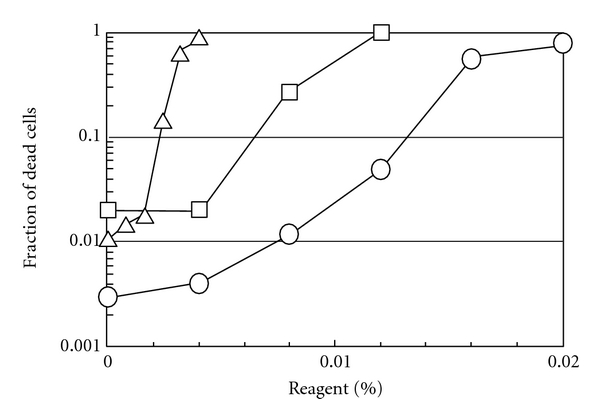
Effect of CAE, BKC, and SDS on the cell viability. Confluent monolayers of Vero cells were incubated in 0.85% sodium chloride solution (pH 5.5) containing 20 mM MES and varying concentrations of each compound at room temperature for 15 min. The treated cells were trypsinized to obtain a single cell suspension and the amounts of the live and dead cells in each culture were determined by a dye exclusion test with trypan blue. Circles (○) for CAE; triangles (△) for BKC; squares (□) for SDS.

The above three compounds were then compared for cell toxicity. As shown in Figure 6, BKC showed a strong cytocidal effect. When the monolayers of Vero cells were incubated in isotonic buffer solution in the presence of various concentrations of CAE, BKC, and SDS for 15 min at room temperature and numbers of dead and alive cells were determined by dye exclusion test with trypan blue, BKC showed a drastic increase in the number of dead cells at the concentration above 0.002%. At the concentration required for the effective virucidal effect (0.003%), BKC killed more than 10% of the cells. In contrast to BKC, cell death by SDS and CAE occurred at 2.5- and 10-fold higher concentrations, indicating their lower cell toxicity. It should be noted that SDS solubilized plasma membrane of the cells at the cytotoxic concentrations while CAE or BKC did not (data not shown), leading to a possible underestimation of the fraction of dead cells in the SDS-treated dishes. The results suggest that at 0.01% CAE, about 2% of the cells were killed by the compound (Figure 6), while more than 99% of the virus was inactivated (Figure 5).

### 3.4. Inhibition of Virus Multiplication by CAE

**Figure 7:**
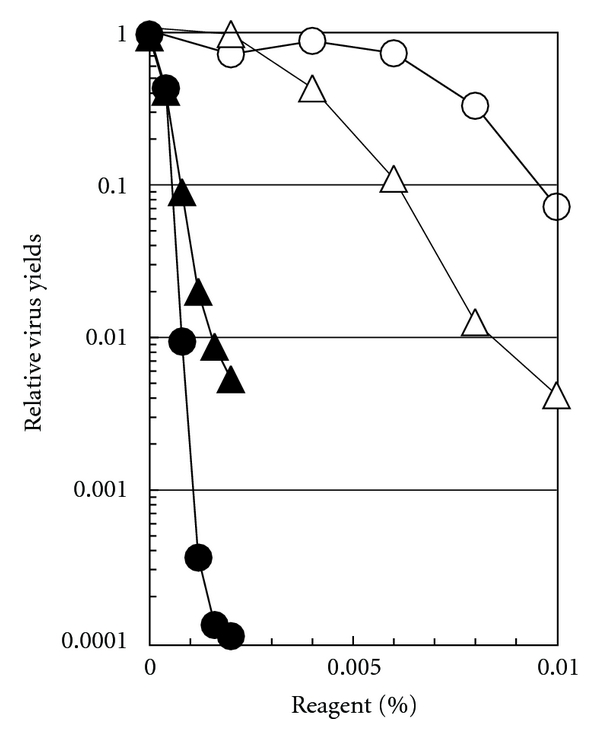
Effect of CAE and BKC on the virus multiplication. Confluent monolayers of HEp-2 cells were infected with HSV-1 (○, ●) at an MOI of 14 or PV-1 (△, ▲) at an MOI of 9. The infected cells were incubated for overnight in the medium containing varying concentrations of CAE (○,△) or BKC (●, ▲) at 37°C for HSV-1 or at 35.5°C for PV-1. At the end of infection (20 h after infection for HSV-1 and 16 h after infection for PV-1), the amounts of infectious progeny viruses were determined and were normalized to the virus yield in the absence of these compounds.

The results in Figures 1–5 show *in vitro* virus inactivation. Whether the same compounds can yield a reduced virus multiplication in *in vivo* experiments was then examined by treating the infected cells with them. Figure 7 shows the results of CAE and BKC on the relative virus yields of HSV-1 (circle) or PV-1 (triangle), when the virus-infected cells were incubated in the medium containing varying concentration of CAE (open symbol) or BKC (closed symbol): note that the highest concentrations used are still below the concentrations used for virus inactivation. The multiplication of these two viruses was much more strongly suppressed by BKC than CAE. However, their effects on these two viruses were different. Namely, CAE suppressed more strongly the virus yield of PV-1 than HSV-1, opposite to BKC. Comparison of the results in Figure 6 suggests that the suppression of these virus multiplications by CEA or BKC was not due to the death of the infected cells. These results show that both BKC and CEA inhibit the multiplication of two viruses widely different in the way the replication and transcription of the viral genome occur (i.e., in the nucleus or in the cytoplasm of the infected cells). It is interesting to note that BKC and CAE, which were both ineffective even at 0.1 and 0.2% in inactivating PV-1, can suppress the growth of the same virus in the infected cells. It is evident that the decreased virus yield in infected cells for PV-1 (and perhaps HSV-1) is not simply due to the virus inactivation.

Generally, virus-inactivating agents (disinfectants) show toxicity to cells and tissues, limiting their applications. We have undertaken a systematic screening of amino acid derived compounds in search for effective, nontoxic virus-inactivating agents and found that, against HSV-1 and HSV-2, CAE has notable activities to inactivate the infectivity of extracellular virus particles and to suppress the virus multiplication in the infected cells at the concentration with tolerable cytotoxic effect. These results may support a future potential application of CAE as a therapeutic or preventive medicine against HSV superficial infection at body surface, such as herpetic keratitis and genital herpes.
